# Controlling heterologous gene expression in yeast cell factories on different carbon substrates and across the diauxic shift: a comparison of yeast promoter activities

**DOI:** 10.1186/s12934-015-0278-5

**Published:** 2015-06-26

**Authors:** Bingyin Peng, Thomas C Williams, Matthew Henry, Lars K Nielsen, Claudia E Vickers

**Affiliations:** Australian Institute for Bioengineering and Nanotechnology (AIBN), The University of Queensland, St. Lucia, QLD 4072 Australia

**Keywords:** Promoter, Overexpression, Metabolic engineering, Batch cultivation, Fermentation, Transcription regulation, Flow cytometry

## Abstract

**Background:**

Predictable control of gene expression is necessary for the rational design and optimization of cell factories. In the yeast *Saccharomyces cerevisiae*, the promoter is one of the most important tools available for controlling gene expression. However, the complex expression patterns of yeast promoters have not been fully characterised and compared on different carbon sources (glucose, sucrose, galactose and ethanol) and across the diauxic shift in glucose batch cultivation. These conditions are of importance to yeast cell factory design because they are commonly used and encountered in industrial processes. Here, the activities of a series of “constitutive” and inducible promoters were characterised in single cells throughout the fermentation using green fluorescent protein (GFP) as a reporter.

**Results:**

The “constitutive” promoters, including glycolytic promoters, transcription elongation factor promoters and ribosomal promoters, differed in their response patterns to different carbon sources; however, in glucose batch cultivation, expression driven by these promoters decreased sharply as glucose was depleted and cells moved towards the diauxic shift. Promoters induced at low-glucose levels (*P*_*HXT7*_, *P*_*SSA1*_ and *P*_*ADH2*_) varied in induction strength on non-glucose carbon sources (sucrose, galactose and ethanol); in contrast to the “constitutive” promoters, GFP expression increased as glucose decreased and cells moved towards the diauxic shift. While lower than several “constitutive” promoters during the exponential phase, expression from the *SSA1* promoter was higher in the post-diauxic phase than the commonly-used *TEF1* promoter. The galactose-inducible *GAL1* promoter provided the highest GFP expression on galactose, and the copper-inducible *CUP1* promoter provided the highest induced GFP expression following the diauxic shift.

**Conclusions:**

The data provides a foundation for predictable and optimised control of gene expression levels on different carbon sources and throughout batch fermentation, including during and after the diauxic shift. This information can be applied for designing expression approaches to improve yields, rates and titres in yeast cell factories.

**Electronic supplementary material:**

The online version of this article (doi:10.1186/s12934-015-0278-5) contains supplementary material, which is available to authorized users.

## Background

The budding yeast *Saccharomyces cerevisiae* is widely used as a cell factory for producing biofuels and biochemicals. Economic application of cell factories requires that feed stocks (carbon sources) are efficiently converted to desired products. Metabolic engineering involves over-expressing certain genes to introduce/enhance/optimize the metabolic network to improve strain performance. The strength and pattern of gene over-expressions are primarily controlled by promoters. Most promoters used in yeast metabolic engineering are endogenous and respond to environmental signals by up-regulation or down-regulation through in vivo transcriptional regulatory networks [1–3]. These networks are subject to change over time with the different cultivation conditions encountered in industrial processes. Predictable modulation of gene expression in cell factory development requires knowledge of the strength and the regulatory pattern of promoters [4, 5].

The carbon source has a significant effect on global regulatory patterns. The effect directly imposed through promoters on the engineered gene expression is of particular importance when developing yeast cell factories [6–8]. When fermentable hexoses (e.g. glucose, fructose) are used as a carbon source, even under aerobic conditions, *S*. *cerevisiae* firstly ferments sugars rapidly into ethanol (the ‘Crabtree effect’) [9]. When the preferred sugar depletes, growth slows while appropriate metabolic networks are switched on for using an alternative carbon source (either the ethanol previously produced or another available carbon source); this phase is known as the ‘diauxic shift’. The cells then continue growth on the alternative substrate(s). To achieve maximum efficiency in the conversion of carbon source to product, it is desirable to achieve good expression levels of the appropriate genes throughout the bioprocess—including during and after the diauxic shift. However, promoter activities during this latter phase of fermentation are not well characterized. Moreover, alternative (non-glucose) carbon sources are becoming recognized as desirable feed stocks [10, 11], and in some cases can provide higher product yields—for example, production of the anti-malaria isoprenoid artemisinin was achieved at higher levels using galactose or ethanol as a carbon source compared to glucose [12]. It is therefore important to consider the expression patterns of commonly-used promoters on other industrially important carbon sources such as sucrose, ethanol, galactose and xylose [8, 13, 14]. Commonly-used promoters are poorly characterized for behavior on these alternative carbon sources.

Commonly-used promoters can be divided into two main classes. ‘Constitutive’ promoters are considered to give stable expression levels across varying culture conditions, while ‘dynamic’ or ‘inducible’ promoters drive dramatic changes in expression level in response to environmental stimuli. Constitutive promoters that drive high level transcription (strong constitutive promoters) are often used for engineering applications. Well-known examples include: promoters of glycolytic genes, such as 3-phosphoglycerate kinase (*P*_*PGK1*_), glyceraldehyde-3-phosphate dehydrogenase (*P*_*TDH3*_), triose phosphate isomerase (*P*_*TPI1*_), enolase (*P*_*ENO*2_) and alcohol dehydrogenase (*P*_*ADH1*_) [3, 5, 15]; and promoters for the genes encoding the cell’s translational machinery, including translational elongation factor EF-1 alpha promoters (*P*_*TEF1*_ and *P*_*TEF2*_), which are thought to enable a relatively stable expression level during glucose batch cultivation [3, 15].

The constitutive expression of certain proteins and metabolic pathways can be detrimental to cell growth due to product toxicity, and the metabolic burden imposed by the redirection of carbon flux, redox cofactors, and ATP [16, 17]. In such scenarios, it is desirable to utilize dynamically regulated promoters to activate a production pathway after a growth phase has been completed [18]. Dynamic control of gene expression can be implemented by using inducible promoters. For example, the *GAL1*/*GAL10* promoter (bidirectional, galactokinase/UDP-glucose-4-epimerase) is induced when cells are grown on galactose [19], and the *CUP1* promoter can be induced by adding copper(II) to a fermentation [20]. Another class of inducible promoters are those which can be induced when glucose is low/absent (low-glucose-inducible), including the high-affinity glucose transporter promoter (*P*_*HXT7*_) [15, 21] and the alcohol dehydrogenase promoter (*P*_*ADH2*_) [5, 22].

Ideally, gene expression would be tailored towards the specific bioprocess and product requirements for metabolic engineering applications, thereby achieving optimal yields/rates/titres [23]. Although previous studies effectively compared the relative strengths of promoters during logarithmic growth on glucose [3, 15, 22], the expression levels on alternative carbon sources such as sucrose, galactose and ethanol, and the promoter activity throughout the time-course of a batch cultivation have not been closely examined. In order to gain an in-depth understanding of promoter performance on different carbon sources and over batch fermentation, we used green fluorescent protein (GFP) to examine the activity of a variety of different promoters. Promoter strengths on glucose, sucrose, galactose and ethanol were characterized, and expression levels over a typical glucose batch cultivation were evaluated. For the *CUP1* promoter, we also characterized the induction profile on a range of different copper concentrations. Our findings reveal the composite activities of various promoters in response to different carbon sources and the diauxic shift.

## Results

### Promoter strength on glucose and comparison of stable and destabilized versions of GFP

A large range of commonly-used promoters were used in addition to a set of novel promoters selected based on transcription profiles. While they have not been investigated previously, ribosomal biogenesis genes account for about 50% of RNA polymerase II transcription in fast growing yeast cells [24]. Thus, their promoters might be useful as strong constitutive promoters for metabolic engineering. Chaperonin *SSA1* gene is up-regulated significantly during growth on ethanol [25, 26] making the *SSA1* promoter a candidate that is automatically induced during the ethanol consumption phase in glucose batch cultivation. The full range of promoters included glycolytic promoters (*P*_*PGK1*_, *P*_*TDH3*_, *P*_*ENO2*_, *P*_*ADH1*_, and *P*_*TPI1*_), translational elongation factor promoters (*TEF*: *P*_*TEF1*_, *P*_*TEF2*_ and *P*_*YEF3*_), galactose metabolic promoters (*P*_*GAL10/GAL1*_), ribosomal protein promoters (*P*_*RPL3*_, *P*_*RPL15A*_, *P*_*RPL4*_ and *P*_*RPL8B*_), chaperone promoters (*P*_*SSA1*_ and *P*_*SSB1*_), the copper-inducible *CUP1* promoter, low-glucose-inducible promoters (*P*_*TPS1*_, *P*_*HXT7*_, *P*_*ADH2*_ and *P*_*CYC1*_), and the *PDA1* promoter (regarded as constitutively expressed [27]). To determine the relative strengths of a range of promoters which are relevant to metabolic engineering we analysed GFP expression levels in single cells from cultures growing exponentially on a range of industrially relevant carbon sources. Minimal media without any added amino acids are used for industrial processes to reduce costs, as well as in metabolic flux analysis to enable accurate quantification of carbon fluxes. To make our promoter activity analysis more relevant to these processes, a yeast classification medium, yeast nitrogen base (YNB) without amino acids, was used to cultivate yeast strains to evaluate each promoter.

**Figure 1:**
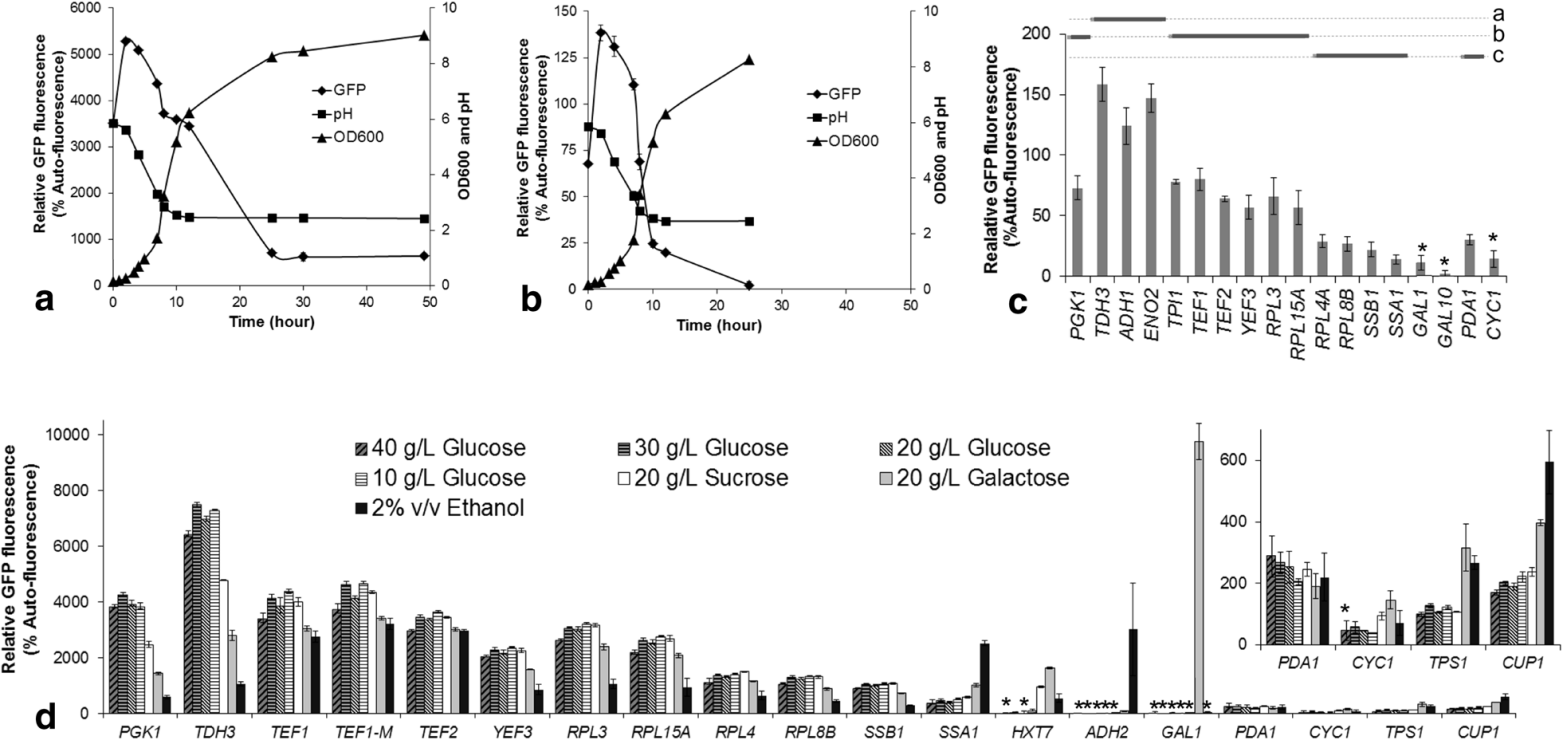
GFP activity driven by different promoters on different carbon sources. GFP fluorescence, culture pH and biomass accumulation (OD_600_) of the *P*_*TEF1*_-yEGFP strain (**a**) and the *P*_*TEF1*_-yEGFP-*CLN2*_PEST_ strain (**b**) in flask batch cultivation in YNB broth with 20 g L^−1^ glucose as the carbon source are shown. GFP fluorescence of various promoter-*yEGFP*-*CLN2*_PEST_ strains on 20 g L^−1^ glucose in microtitre plate culture (**c**) and of various promoter-*yEGFP* strains on various carbon sources in microtitre plate culture (**d**) are also shown. The GFP fluorescence in (**c**) was ranged using Tukey’s test: three levels were identified (*dashed lines* *a* > *b* > *c*) within which the difference between group members (*bold lines*) was insignificant (*p* > 0.05). In *TEF1*-*M* (**d**) construction, A *Xho*I site plus triple “A” was inserted between *TEF1* promoter and the start codon of *yEGFP*. The insert in (**d**) shows a zoomed-in GFP fluorescence scale for the weaker promoters, *P*_*PDA1*_, *P*_*CYC1*_, *P*_*TPS1*_ and *P*_*CUP1*_. The analysis of variance for the fluorescence levels in (**d**) is shown in Additional file 1: Figure S3. The auto-fluorescence was determined from the reference strains (ILHA GH4 for the yEGFP strains and ILHA GFP3 for the *yEGFP*-*CLN2*_PEST_ strains) in parallel. Symbol *Asterisk* represents that the value is <50 and not significantly different from auto-fluorescence (*t* test, *p* > 0.05). Mean values ± standard deviations are shown from replicate cultivations.

The yeast enhanced green fluorescent protein (*yEGFP*) and a destabilized version, *yEGFP*-*CLN2*_*PEST*_ (*yEGFP* fused with the G1 cyclin PEST sequence, a peptide sequence rich in proline, glutamic acid, serine, and threonine that causes protein destabilization), were used as reporters to compare promoter expression levels. Expression of *yEGFP* reflects the accumulation level of a stable protein with a half-life of ~7 h, while the destabilized GFP shows the dynamic protein synthesis rate, because of its short half-life (12 min) [28]. Using *TEF1* promoter for pre-evaluation, we observed that for both *yEGFP* and *yEGFP*-*CLN2*_*PEST*_ reporter genes, the intracellular GFP levels varied throughout the cultivation time (Figure 1a, b). In order to minimize the discrepancies caused by difference in the culture state during subsequent high-throughput microtitre plate analysis, GFP measurements were taken when OD_600_ ranged from 1 to 2.5 (mid-log phase; see Additional file 1: Figure S1).

A set of promoters was initially tested on 20 g L^−1^ glucose using the destabilised GFP in microtitre plate format (Figure 1c). *P*_*TDH3*_, *P*_*ENO2*_ and *P*_*ADH1*_ were the three strongest promoters during mid-log phase, followed by *P*_*PGK1*_, *P*_*TPI1*_, *P*_*TEF1*_, *P*_*TEF2*_, *P*_*YEF3*_, *P*_*RPL3*_ and *P*_*RPL15A*_*; P*_*RPL4A*_, *P*_*RPL8B*_, *P*_*PDA1*_, *P*_*SSB1*_ and *P*_*SSA1*_were weaker promoters. In subsequent experiment, we found that the signal-to-noise ratio using destabilized GFP assay was too low to reasonably compare promoter strength on some carbon sources (Additional file 1: Table S1). Therefore, the standard GFP (without *CLN2*_*PEST*_) was used as reporter to compare the promoter activities on different carbon sources. While GFP levels from the stable yEGFP were ~50-fold higher than that from the destabilized yEGFP, the GFP levels were highly correlated (*R*^2^ = 0.98, 11 df, *p* = 7.69 × 10^−8^, Additional file 1: Figure S2), indicating that stable GFP can be used to reproducibly report relative promoter activity despite the longer protein half-life.

An expanded set of promoters, including most of the promoters in the analysis using the destabilised GFP, were tested using the standard GFP on a range of different carbon sources as well as varying glucose concentration in the medium (40, 30, 20, and 10 g L^−1^) (Figure 1d). Varying the glucose level had only a minor effect on GFP activity for each promoter with no clear patterns apparent (Figure 1d; Additional file 1: Table S2).

In conventional genetic cloning, a restriction site is commonly introduced between the promoter and the ATG start codon. This can potentially interfere with transcription/translation. To examine this, we introduced a *Xho*I site plus triple “A” between the *TEF1* promoter and the start codon. No significant influence in GFP level was observed on glucose or on any of the other carbon sources (Figure 1d, TEF1-M strain; two-way anova, F_6, 28_ = 0.19, *p* = 0.98).

### Promoter response patterns on different carbon sources

Promoter activity was tested on media containing 20 g L^−1^ sucrose, 20 g L^−1^ galactose and 2% v/v ethanol (Figure 1d). A comparison of GFP activities between cells grown on glucose and sucrose showed that most promoter classes, including the translational elongation factor (*P*_*TEF1*_, *P*_*TEF2*_ and *P*_*YEF3*_), ribosomal (*P*_*RPL3*_, *P*_*RPL15A*_, *P*_*RPL4*_ and *P*_*RPL8B*_), galactose-responsive (*P*_*GAL1*_), chaperone (*P*_*SSA1*_ and *P*_*SSB1*_), copper-responsive (*P*_*CUP1*_) and *PDA1* promoters showed no difference in GFP levels between glucose and sucrose. The ‘low-glucose-inducible’ promoters had varying responses: *P*_*TPS1*_ (low levels on glucose) also showed no difference on sucrose, whereas *P*_*CYC1*_ showed a slight increase on sucrose, and *P*_*HXT7*_ (which was weak under all of the glucose concentrations examined) was de-repressed on sucrose, resulting in an intermediate level of activity (relative to activity of other promoters on sucrose). The glycolytic promoters (*P*_*PGK1*_ and *P*_*TDH3*_) showed decreased levels of GFP activity on sucrose relative to glucose (by 37 and 32%, respectively).

As expected, the *GAL1* promoter exhibited the highest activity on galactose (Figure 1d). The glycolytic promoters, *TEF* promoters, ribosome promoters, and the *SSB1* promoter all showed decreased GFP activity to varying degrees on galactose relative to both sucrose and glucose (Figure 1d). An increase in activity on galactose by about 1–2 fold was observed for the *TPS1*, *SSA1* and *CUP1* promoters.

The *ADH2*, *TEF1*, *TEF2*, and *SSA1* promoters were the four strongest promoters during growth on ethanol, and all showed similar levels of activity (Figure 1d). The strengths of *P*_*TEF1*_ and *P*_*TEF2*_ were similar on ethanol as on galactose (i.e. lower than on glucose); *P*_*SSA1*_ was about ~5-fold higher relative on ethanol to 20 g L^−1^ glucose. The *ADH2* promoter was repressed on most carbon sources; it was strongly induced on ethanol, and also showed low level activity on galactose. The *HXT7* promoter was up-regulated on ethanol, but the GFP level was lower than on sucrose and galactose. The *TPS1*, *CYC1* and *CUP1* promoters were up-regulated on ethanol compared to glucose/sucrose, but were still relatively weak. Activity driven by the *TDH3* and *PGK1* promoters was low on ethanol; it was reduced by ~85% relative to activity on 20 g L^−1^ glucose and by ~60% relative to 20 g L^−1^ galactose. Activity driven by the ribosomal promoters, the *YEF3* promoter, and the *SSB1* promoter was more than 50% reduced compared to activity on 20 g L^−1^ glucose (Figure 1d).

*PDA1* is reported as being constitutively expressed on different carbon sources [27]. Consistent herewith, GFP expression from the *PDA1* promoter on the seven different conditions did not vary significantly (Figure 1d, one-way ANOVA, F_6, 14_ = 1.62, *p* = 0.21). However, compared to other promoters investigated, the activity level was relatively low.

### Promoter performance over the diauxic shift in glucose batch cultivation

**Figure 2:**
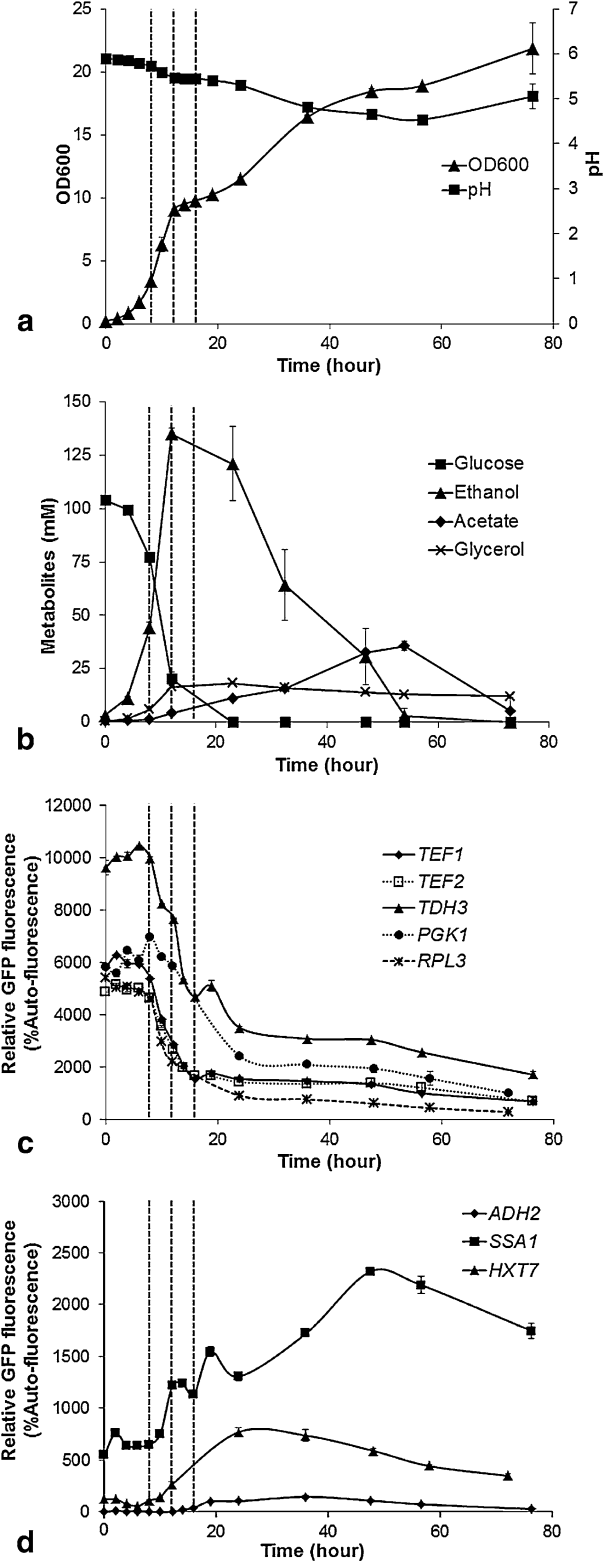
Promoter performance over the diauxic shift: **a** cell growth and pH of the reference strain ILHA GH4; **b** the profile of extracellular metabolites. **c** the fluorescence of GFP controlled by promoters classically considered to be “constitutive”; **d** the fluorescence of GFP controlled by low-glucose-inducible promoters. The *vertical dash lines* are at 8, 12 and 16 h. The auto-fluorescence was determined from the reference strain in parallel. The medium was buffered with 100 mM MES. Mean values ± standard deviations are shown from duplicate cultivations.

Glucose batch cultivation involves a diauxic shift from glucose respire-fermentative metabolism to ethanol respiratory metabolism (Figure 2a, b). The diauxic shift and the ethanol consumption phase which follows can be far longer than the initial exponential growth phase in some industrial yeast bioprocesses. A thorough understanding of the expression levels of commonly used promoters throughout and after the diauxic shift is therefore necessary to predict and optimise the productivity of yeast cell factories. In order to explore promoter performance over the diauxic shift, several promoters with high strength on glucose and/or ethanol (*P*_*TEF1*_, *P*_*TEF2*_, *P*_*TDH3*_, *P*_*PGK1*_, *P*_*RPL3*_, *P*_*SSA1*_, *P*_*ADH2*_ and *P*_*HXT7*_; Figure 1c, d) were compared using time-course measurements of GFP expression levels throughout a typical glucose batch cultivation. Because of the weak pH-buffer capacity of commercial YNB media (Figure 1a, b), 100 mM 4-morpholineethanesulfonic acid (MES) buffer was added to maintain relatively stable pH during flask cultivation and prevent pH-based growth limitation (Figure 2a).

In batch cultivation where cultures were inoculated with cells in mid-log phase, changes in cellular GFP level began after 8 h (Figure 2c, d), approximately when the diauxic shift occurred, before glucose became depleted and the cells shifted from using glucose to using ethanol (Figure 2a, b). GFP level driven by the *TEF1*, *TEF2*, *TDH3*, *PGK1* and *RPL3* promoters decreased dramatically, and kept decreasing sharply until 16–20 h (Figure 2c). GFP levels were then maintained at relatively low levels during the ethanol respiratory phase, further decreasing when ethanol was completely depleted (Figure 2b, c). This profile differed somewhat to that observed when ethanol was used as the carbon source in batch culture, where GFP levels driven by *P*_*TEF1*_ and *P*_*TEF2*_ were higher than the levels driven by *P*_*TDH3*_ and *P*_*PGK1*_ (Figure 1d). The relative GFP expression levels during the ethanol consumption phase of the batch culture (Figure 2c) were:

*P*_*TDH3*_ > *P*_*PGK1*_ > *P*_*TEF1*_ ~ *P*_*TEF2*_ > *P*_*RPL3*_

In contrast, the remaining three low-glucose-inducible/ethanol-responsive promoters showed an increase in GFP activity upon the diauxic shift. The increase in GFP expression from the *SSA1* and *HXT7* promoters began at 8 h, and peaked at 48 and 24 h, respectively (Figure 2d). This is consistent with the observation from the ethanol batch cultivation (Figure 1d) showing that the *SSA1* and *HXT7* promoters are induced on ethanol. Surprisingly, and in contrast to the ethanol batch cultivation (Figure 1d), the *ADH2* promoter was not induced to a considerably high level during the post-diauxic shift ethanol consumption phase; GFP activity was about 30-fold lower in the glucose-ethanol batch cultivation, (relative GFP fluorescence of approximately 100 vs. 3,000: Figure 2c compared to Figure 1d).

### Induction of the *CUP1* promoter under varying copper concentrations

**Figure 3:**
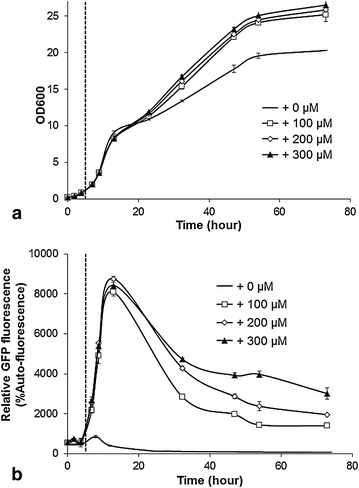
Copper induction of the *CUP1* promoter: **a** population growth of the reference strain ILHA GH4; **b** *CUP1* promoter-regulated GFP activity. 1 M copper sulphate was added to final concentrations of 0, 100, 200 or 300 μM at 5 h (*vertical dash line*). The auto-fluorescence was determined from the reference strain in parallel. The medium was buffered with 100 mM MES. Mean values ± standard deviations from duplicate cultivations are shown.

Inducible promoters enable the up-regulation of gene expression via the addition of an inducer chemical at the desired processing time. The *CUP1* promoter can be induced by copper, which is not prohibitively expensive for use in an industrial setting. The *CUP1* promoter was leaky in the absence of copper(II), although this level of expression was weaker by an order of magnitude than the strong constitutive promoters (e.g. *P*_*TEF1*_; Figure 1d). To test the *CUP1* promoter strength under different inducing conditions, different concentrations of copper were added to exponentially growing populations (Figure 3). The addition of copper resulted in significantly higher OD_600_ readings after 20 h of growth (Figure 3a), most likely due to the formation of a ‘rusty’ colour in cells (Additional file 1: Figure S4) that might interfere with OD_600_ readings after copper addition.

When the *CUP1* promoter was induced with 100, 200 or 300 μM copper (Figure 3b), the maximum strength of the *CUP1* promoter after induction was lower than the *TDH3* promoter but higher than the *TEF1* promoter during the exponential phase (Figure 3b). Although GFP activity decreased after the diauxic shift, there was a positive correlation with the copper concentration. With 300 μM copper induction the *CUP1* promoter resulted in the highest post-exponential-phase GFP level (Figure 3).

## Discussion

In the rational design and optimization of metabolic pathways for the development of yeast cell factories, promoters are currently the most important tool available for controlling gene expression. It is therefore necessary to fully understand promoter strength and expression patterns across industrially relevant conditions. Promoters are known to vary in activity on different carbon sources and over time throughout fermentations [15, 29]. Therefore, we analysed the expression levels of a series of constitutive or inducible promoters with the consideration of several important parameters relevant to industrial processes, including carbon source, diauxic shift, and inducible expression.

A ‘promoter’ sequence can be loosely defined as the upstream region of a gene that can replicate the observed expression pattern of that gene when fused to a reporter gene with an easily-assayable phenotype. Several reporter genes are available, and each has different characteristics that make it suitable for different applications [30, 31]. The length of the promoter (bp) is not necessarily well defined or conserved between experimental systems, unless extensive deletion/mutation analysis has been performed using reporter genes [32–34]. Variable promoter lengths can have a significant effect on activity due to presence/absence of *cis*-acting elements within the sequence. We selected a ~450-to-750 bp sequence upstream of each gene (see Additional file 1: Table S3) to test for functionality in batch culture. These promoters were amplified directly from *S*. *cerevisiae* CEN.PK genomic DNA and fused to either a stabilised or a destabilised GFP reporter gene. Promoter activities were tested on a variety of different carbon sources using single-copy genomic insertion strains and flow cytometry-based single-cell GFP activity analysis. The destabilized GFP (~12 min half-life) provides an instantaneous readout of promoter activity, while the stable GFP integrates activity with a protein half-life of ~7 h [28]. However, the responses were highly correlated (Additional fie 1: Figure S2) and the latter provided a far better signal-to-noise ratio for some of the carbon sources.

The advantage of the commonly used *TEF1* and *TEF2* promoters is to enable stable, high-level expression on different carbon sources (Figure 1d). However, we found that *TEF* promoter activity significantly decreased over the diauxic shift, as did activity driven by the *TDH3*, *PGK1* and *RPL3* promoters (Figure 2c). This is consistent with reports that the depletion of glucose blocks translation [35–37]. In contrast to our observations, Sun et al. [3] observed stable GFP florescence driven by the *TEF1* promoter over the diauxic shift. This may be due to differences between media, and/or background strain, and/or differences in the promoter sequences (which were amplified from different strains). Sun et al. used synthetic complete media with added amino acids, whereas here we used a defined medium without added amino acids. Amino acids might activate the TOR (target of rapamycin) signalling pathway, thereby interfering with the translational blockage phenotype [38, 39] which is regulated by the depletion of glucose. Our observations can potentially explain the weak productivity of cell factories during the ethanol consumption phase in minimal media when *TEF1* and *PGK1* promoters are used to control production pathway gene expression [40].

Another important consideration in metabolic engineering is that high-level expression of certain pathways might result in detrimental effects on cell growth [18]. In this regard, the *SSA1* promoter could be used to automatically increase the expression of production pathways as biomass increases (Figure 2). Up-regulation of the *SSA1* promoter can be attributed to its heat shock transcription factor Hsf1p-mediated transcription [25, 26, 36]. Similarly, the up-regulation of *CUP1* promoter on ethanol (Figure 1d) is also mediated by Hsf1p which is activated by Snf1p, a protein kinase required by the expression of glucose-repressing genes [41]. Consistent with this idea, another Hsf1p-mediated promoter (*HSP26* promoter) has been shown to improve the performance of cell factories in glucose-depleted conditions [36, 42].

GFP expression from the *HXT7* promoter also gradually increased after the diauxic shift, at high biomass levels (Figure 2d). However, in contrast with previous reports [15, 21], it did not show very high activity; GFP levels were comparable to the *TEF1* promoter in the same phase of the culture. As noted above, differences might be due to variation in promoter sequence (the promoter used here is 80 bp longer than the previously-analysed promoter [15]).

The *ADH2* promoter showed the strongest activity on ethanol when provided with ethanol as a carbon source (Figure 1d). However, this promoter was not fully de-repressed after the diauxic shift in glucose batch cultivation (Figure 2d), despite being rapidly de-repressed after inoculating ethanol-phase cells from glucose batch cultures into fresh YNB ethanol media (Additional file 1: Figure S5). Lee and DaSilva [22] showed that *ADH2* can be fully de-repressed in complete media (containing yeast extract and peptone) but not in minimal media. This is consistent with our data (Figure 2d) considering that minimal medium with an excess of ammonium was used. Combined with the rapid de-repression on fresh YNB ethanol media, it suggests that a secondary, currently un-elucidated mechanism that is not related to nitrogen source, might be involved in the de-repression of the *ADH2* promoter. Regardless, in glucose batch culture the *ADH2* promoter may not be ideal for driving high-level expression after the diauxic shift due to its low expression level in the ethanol phase in batch cultivation. However, it is applicable as a fine genetic switch to achieve low-level expression as cells shift to the ethanol phase, because it is fully repressed on the glucose phase (Figure 2d).

Aside from investigating low-glucose-inducible promoters for dynamic regulatory patterns, we also characterized the copper-inducible *CUP1* promoter. A very high level of GFP expression was achieved by using the *CUP1* promoter and copper(II) feeding (Figure 3). However, after peaking, activity from the *CUP1* promoter decreased sharply. This might be caused by the copper(II) detoxification mechanism in yeast whereby cells can reduce copper(II) to copper(I) [43], and copper (I) can be bound by metallothionein (Cup1p) [43, 44] or automatically converted to copper metal through disproportionation. This mechanism might essentially remove the inducing agent from the medium, resulting in the sharp decline in GFP activity. Even considering its decreased strength after peaking, the *CUP1* promoter is still superior to other promoters because it enables inducible, and high-level GFP expression after the diauxic shift (Figure 3)—a pattern which might be useful for specific applications where high-level expression of certain genes causes the delayed cell growth and/or alternative carbon source like ethanol needs to be consumed to maximize the production yield/titre.

## Conclusions

Different carbon sources and the diauxic shift can influence gene expression driven by endogenous promoters, resulting in different response patterns. The analysis of promoter-regulated gene expression presented here can be used to inform the rational design of metabolic pathways and synthetic genetic circuits, and reveals potential limitations in current metabolic engineering strategies. In particular, the relatively low expression levels observed for all of the ‘constitutive’ promoters after the diauxic shift during glucose batch cultivation suggests a novel target for metabolic engineering in yeast and the expression patterns of low-glucose-inducible or copper-inducible promoters reveals potential utility for the dynamic regulation of synthetic genetic/metabolic networks. Our results provide instructive and in-depth information about promoter performance on different carbon sources and during the diauxic shift, and can be used to inform expression pattern design for yeast cell factories. It would be useful to examine promoter responses on other industrially-useful carbon sources—in particular, cellulosic feedstock sugars such as xylose, which yeast have been successfully engineered to utilize [8, 45].

## Methods

### Plasmid construction and strain construction

**Figure 4:**
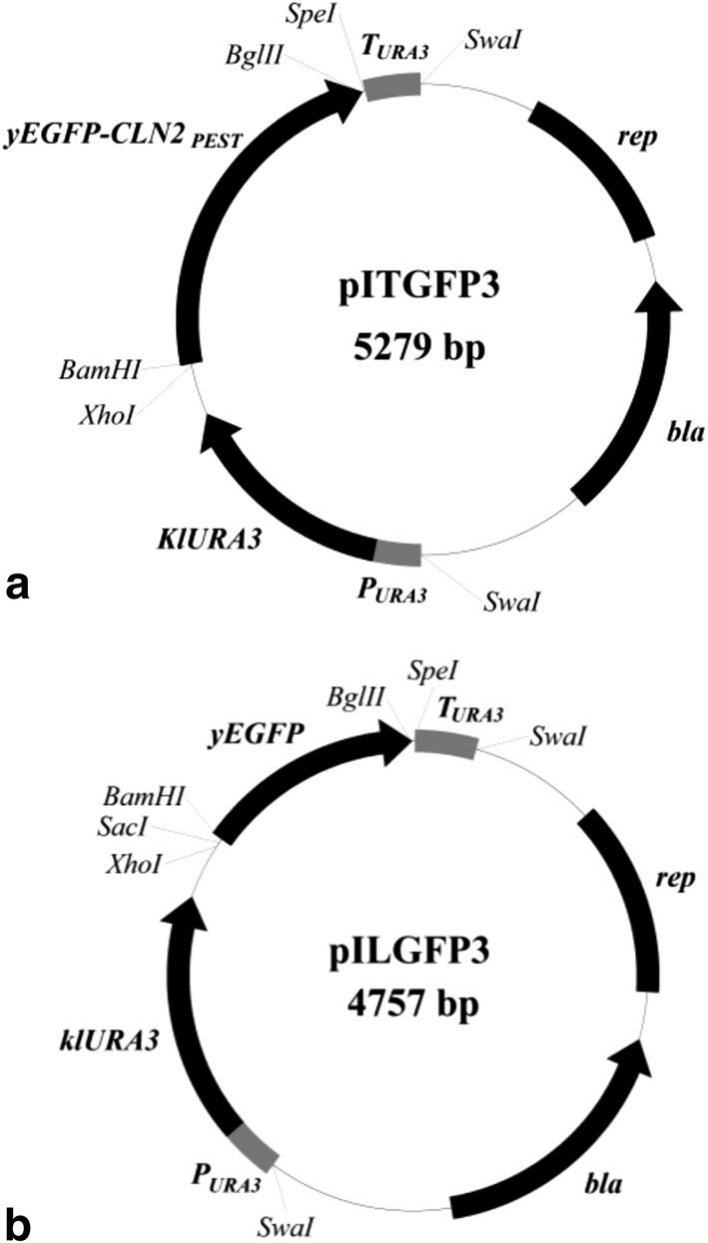
Physical maps of plasmids pITGFP3 (**a**) and pILGFP3 (**b**): *rep* pUC19 replicon in *E*. *coli*, *bla* ampicillin resistant gene in *E*. *coli*, *P*_*URA3*_ *URA3* promoter of *S*. *cerevisiae*, *KlURA3* *Kluyveromyces* *lactis* *URA3* gene, *yEGFP* yeast enhanced green fluorescence gene, *CLN2*_*PEST*_ encoding the protein-destabilizing peptide from cyclin 1 of *S*. *cerevisiae*, *T*_*URA3*_ *URA3* terminator of *S*. *cerevisiae*.

Primers, plasmids, strains, promoters and promoter lengths used in this study are listed in Additional file 1: Table S3. The *URA3* promoter (*P*_*URA3*_) and *URA3* terminator (*T*_*URA3*_) were amplified from CEN.PK113-7D [46] genomic DNA; *KluyveromyceslactisURA3* (*KlURA3*) was amplified from plasmid pUG72 [47]; and yeast enhanced green fluorescent protein (*yEGFP*) and *yEGFP*-*CLN2*_PEST_ sequence were amplified from pFA6a-yEGFP3-CLN2_PEST_-natMX6 [48]. The fragment *P*_*URA3*_- *KlURA3*- *yEGFP3*- *CLN2*_PEST_- *T*_*URA3*_ was fused together through overlap extension PCR and cloned into *Sph*I/*Eco*RI sites of pUC19 [49] to generate the plasmid pITGFP3 (Figure 4a). The *yEGFP* fragment was cloned into *Xho*I/*Spe*I sites of pITGFP3 to replace *yEGFP3*- *CLN2*_PEST_ and generate the plasmid pILGFP3 (Figure 4b). On plasmids pITGFP3 and pILGFP3, the restriction site *Bam*HI was present 3′ of the initial start codon of *yEGFP* to eliminate the influence of (−3, −1) region on promoter strength [50]. pILGFP3 was digested with *Bam*HI/*Bgl*II and self-ligated to generate the control plasmid pILGH4 (without GFP). The tested promoters (Additional file 1: Table S3) were amplified from *S*. *cerevisiae* CEN.PK113-7D [46] genomic DNA. All promoters were digested by *Xho*I/*Bam*HI and cloned into *Xho*I/*Bam*HI sites to generate promoter-testing plasmids (Additional file 1: Table S3). The control plasmids (pITGFP3 and pILGH4) and the promoter-testing plasmids were digested with *Swa*I to linearize, and transformed into CEN.PK113-5D [46] to generate reference strains (ILHA GPP3 and ILHA GH4) and promoter-testing strains (Additional file 1: Table S3). PCR and sequencing was performed to verify the transformants and select strains containing single-copy integration at the *ura3* locus. The recombinant strains were stored as glycerol stocks at −80°C.

### Flask cultivation

The minimal media used to analyse the GFP expression level contain 6.7 g L^−1^ YNB pH6.0 (Sigma-Aldrich). YNB with 20 g L^−1^ glucose (YNBG) was used as the medium without additional buffer. To buffer against the significant changes in pH, 100 mM MES was supplied in YNBG and the pH was initially adjusted to 6.0 with ammonium hydroxide. The tested strains were recovered from glycerol stocks by streaking on the YNBG agar plates. A single colony was used to initiate a seed culture by inoculating into 5 ml YNBG broth (or YNBG with 100 mM MES buffer) to a cell density (OD_600_) of 0.02. Over-night, seed cells in the mid-exponential phase (OD_600_ = 1.5–3) were inoculated into 20 mL of YNBG (or YNBG with 100 mM MES buffer) in a 125 mL flask to a starting OD_600_ of 0.2, and incubated at 30°C at 200 rpm. To examine copper induction of the *CUP1* promoter, YNBG with 100 mM MES buffer was used as the medium and copper(II) sulfate stock solution (1 M) was added to the 20 mL flask cultures at 5 h. Cultures were periodically sampled for the measurement of OD_600_, GFP fluorescence, pH, and/or extracellular metabolites. Each strain was cultivated in duplicate.

### Microplate cultivation

To test promoter activity in a high-throughput format on different carbon sources, microplate cultivation was performed at 30°C in a 350 rpm shaking incubator using a U-bottom 96-well plate (Costar#3799, USA) sealed with a Breathe-Easy sealing membrane (Sigma-Aldrich#Z380059, USA). Each strain was cultivated in triplicate for testing. The tested strains were recovered from glycerol stocks by streaking on the YNBG agar plates. To prepare the seed culture, a single colony was resuspended in 100 μL YNBG broth, and 5 μL of resuspended cells were inoculated in 95 μL fresh YNBG (pre-culture 1). The seed cultures were cultivated overnight.

For the analysis using the destabilized GFP (y*EGFP*-*CLN2*_*PEST*_), 5 μL overnight pre-culture 1 was inoculated into 95 μL YNBG in a fresh plate (pre-culture 2), and 5.5 h later, 5 μL culture was inoculated into 95 μL fresh YNBG in a fresh plate (test culture). After 6.5 h, the cells were analysed for GFP fluorescence (see below).

For the analysis using the normal y*EGFP*, pre-culture 1 was firstly diluted 10-fold in a fresh broth of YNB without a carbon source, and then 1 μL diluted culture was inoculated into 100 μL YNB broth with the either 40 g L^−1^ glucose, 30 g L^−1^ glucose, 20 g L^−1^ glucose, 10 g L^−1^ glucose, 20 g L^−1^ sucrose, or 20 g L^−1^ galactose. For the ethanol carbon source (where strains grew much more slowly), 2.5 μL of pre-culture 1 was inoculated into 97.5 μL YNB broth with 2% (v/v) ethanol. After 24 h this dilution was repeated to inoculate the test culture. Cells in mid-exponential phase (OD_600_ = 1–2.5) were analysed for GFP level (see below).

### GFP fluorescence determination

GFP fluorescence in single cells was analysed, immediately after sampling, using a flow cytometer (BD Accuri™ C6; BD Biosciences, USA). GFP fluorescence was excited by a 488 nm laser and monitored through a FL1.A filter (wavelength 530/20 nm). For plate cultures, 5,000 events were counted; for flask cultures, 10,000 events were counted. The particle volume and complexity for each event were monitored by forward scatter detector (FSC.A) and side scatter detector (SSC.A). For flask cultures, the cells were diluted fivefold with water when the OD_600_ was above 10.

The GFP fluorescence signal was corrected for cell size and complexity using a heuristic formula,

$${\text{NormFL}}1.{\text{A}} = {\text{FL}}1.{\text{A}} \times \sqrt {\frac{1}{{{\text{FSC}}.{\text{A}} \times {\text{SSC}}.{\text{A}}}}}$$

where FL1.A, FSC.A and SSC.A are the mean values of 5,000 or 10,000 events. Normalization was particularly important for cells grown in galactose-based medium. Results were expressed as fluorescence relative to auto-fluorescence for the reference strain (ILHA GPP3 or ILHA GH4) cultivated under identical conditions,

$$\begin{array}{*{20}c} {\text{The relative GFP fluorescence}} \\ {\left( {{\text{\% auto-fluorescence}}} \right)} \\ \end{array} = \left( {\frac{{{\text{NormFL}}1.{\text{A}}}}{{{\text{Ref}}\_{\text{NormFL}}1.{\text{A}}}} - 1} \right) \times 100$$

### Extracellular metabolite analysis

Extracellular metabolites (glucose, ethanol, acetate and glycerol) were analysed by Metabolomics Australia Queensland Node through ion-exclusion chromatography [51]. Ion-exclusion chromatography was performed using an Agilent 1200 HPLC system and an Agilent Hiplex H column (300 × 7.7 mm, PL1170-6830) with guard column (SecurityGuard Carbo-H, Phenomenex PN: AJO-4490). Analytes were eluted isocratically with 4 mM H_2_SO_4_ at 0.6 mL/min at 65°C. Glucose, ethanol and glycerol were monitored using a refractive index detector (Agilent RID, G1362A), and acetate was detected using an ultraviolet–visible light absorbance detector (Agilent MWD, G1365B) at 210 nm.

### Statistical analysis

All statistical analyses were performed in R. The relative fluorescence (NormFL.A/Ref_NormFL1.A) data were log transformed, in order to stabilise variance across the dataset. A Bartlett test was used to confirm variance homogeneity for each dataset prior to performing linear regression or analysis of variance (ANOVA). Post-hoc analyses for one-way ANOVA were performed using Tukey tests. In the few instances where data failed the Bartlett test for variance homogeneity, the one-way ANOVA was performed using Welch corrections and posthoc analysis was performed with a Games–Howell test instead of a Tukey test.

## Additional file

Additional file 1:
**Table S1**. Fluorescence of the destabilized GFP (*yEGFP-CLN2*_PEST_) in the strains cultivated on 2% v/v ethanol to OD_600_ = 0.90 ± 0.03. **Table S2**. Effect of difference glucose concentrations (linear regression) and different carbon sources (one-way ANOVA) on GFP fluorescence driven by various promoters. **Table S3**. The primers, the plasmids and the strains used in this work. **Figure S1**. Pre-evaluation of mid-log phase for microplate cultivation. **Figure S2**. Correlation of GFP fluorescence determination in the strains using either the destabilized GFP (*yEGFP-CLN2*_PEST_) or the normal GFP (*yEGFP*) as the reporter. **Figure S3**. Post-hoc test for fluorescence levels (sorted from low to high) of various promoter-*yEGFP* strains on different carbon source. **Figure S4**. Yeast cultures with/without copper addition. **Figure S5**. De-repression of *ADH2* promoter.

## Abbreviations

*P*_*XXXN*_: the promoter of the gene, *XXXN*
GFP: green fluorescence protein
yEGFP: yeast enhanced green fluorescence protein
PEST: a peptide sequence rich in proline, glutamic acid, serine, and threonine
