# Supplementary material for: Controlling heterologous gene expression in yeast cell factories on different carbon substrates and across the diauxic shift: a comparison of yeast promoter activities
Source: Microb Cell Fact. 2015 Jun 26;14:91. doi: 10.1186/s12934-015-0278-5 (PMC4480987; doi:10.1186/s12934-015-0278-5)
Supplement: Additional file 1: — Table S1. Fluorescence of the destabilized GFP (yEGFP-CLN2 PEST) in the strains cultivated on 2% v/v ethanol to OD600 = 0.90 ± 0.03. Table S2. Effect of difference glucose concentrations (linear regression) and different carbon sources (one-way ANOVA) on GFP fluorescence driven by various promoters. Table S3. The primers, the plasmids and the strains used in this work. Figure S1. Pre-evaluation of mid-log phase for microplate cultivation. Figure S2. Correlation of GFP fluorescence determination in the strains using either the destabilized GFP (yEGFP-CLN2 PEST) or the normal GFP (yEGFP) as the reporter. Figure S3. Post-hoc test for fluorescence levels (sorted from low to high) of various promoter-yEGFP strains on different carbon source. Figure S4. Yeast cultures with/without copper addition. Figure S5. De-repression of ADH2 promoter. [file 12934_2015_278_MOESM1_ESM.docx]

**Additional file 1**

Supplementary materials for:

Controlling heterologous gene expression in yeast cell factories on different carbon substrates and across the diauxic shift: a comparison of yeast promoter activities

Authors

Bingyin Peng: Australian Institute for Bioengineering and Nanotechnology (AIBN), The University of Queensland, St. Lucia, QLD 4072, Australia; [bingyin.peng@uq.net.au](mailto:bingyin.peng@uq.net.au)

Thomas C. Williams: Australian Institute for Bioengineering and Nanotechnology (AIBN), The University of Queensland, St. Lucia, QLD 4072, Australia; [thomas.williams1@uqconnect.edu.au](mailto:thomas.williams1@uqconnect.edu.au)

Matthew Henry: Australian Institute for Bioengineering and Nanotechnology (AIBN), The University of Queensland, St. Lucia, QLD 4072, Australia; [matthew.henry2@uq.net.au](mailto:matthew.henry2@uq.net.au)

Lars K. Nielsen: Australian Institute for Bioengineering and Nanotechnology (AIBN), The University of Queensland, St. Lucia, QLD 4072, Australia; [lars.nielsen@uq.edu.au](mailto:lars.nielsen@uq.edu.au)

Claudia E. Vickers*: Australian Institute for Bioengineering and Nanotechnology (AIBN), The University of Queensland, St. Lucia, QLD 4072, Australia; [c.vickers@uq.edu.au](mailto:c.vickers@uq.edu.au)

*, the corresponding author

**Additional file 1: Table S1:** Fluorescence of the destabilized GFP (yEGFP-*CLN2*_PEST_) in the strains cultivated on 2% v/v ethanol to OD_600_= 0.90±0.03

| Promoter | The relative GFP fluorescence (% Auto-fluorescence) |
| --- | --- |
| *PGK1* | 2±3 |
| *TDH3* | 5±7 |
| *ADH1* | 3±10 |
| *ENO2* | 2±4 |
| *TPI1* | 6±6 |
| *GAL1* | 2±11 |
| *YEF3p* | 8±12 |
| *TEF1p* | 32±16 |
| *TEF2p* | 22±13 |
| *PDA1p* | 5±3 |
| *CYC1p* | 23±20 |
| *RPL4A* | 1±2 |
| *RPL15A* | 16±7 |
| *RPL8B* | 4±3 |
| *RPL3* | 17±14 |
| *SSA1* | 4±2 |
| *SSB1* | 4±2 |

The auto-fluorescence was determined from the reference strain on parallel; the value is represented as mean ± standard derivation in the triplicate cultivations.

**Additional file 1: Table S2:** Effect of difference glucose concentrations (linear regression) and different carbon sources (one-way ANOVA) on GFP fluorescence driven by various promoters.

1. Glucose concentrations of 30 g L^-1^, 20 g L^-1^ and 10 g L^-1^.
2. Glucose concentrations of 40 g L^-1^, 30 g L^-1^, 20 g L^-1^ and 10 g L^-1^.
3. Carbon sources: 20 g L^-1^ glucose, 20 g L^-1^ sucrose, 20 g L^-1^ galactose and 2% v/v ethanol

**Additional file 1: Table S3:** The primers, the plasmids and the strains used in this work

- pITGFP__ plasmids contain the expression cassette of the destabilized GFP, yEGFP-CLN2PEST; pILGFP__ plasmids contain the expression cassette of the normal yEGFP.
- ILHA G__ strains (except of ILHA GH4) contain the expression cassette of the destabilized GFP, yEGFP-CLN2PEST; ILHA G__S strains contain the expression cassette of the normal yEGFP.


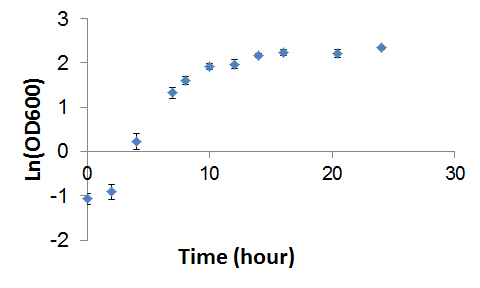

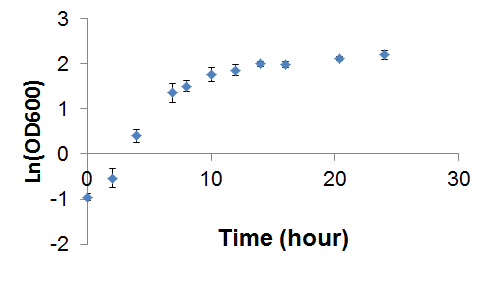

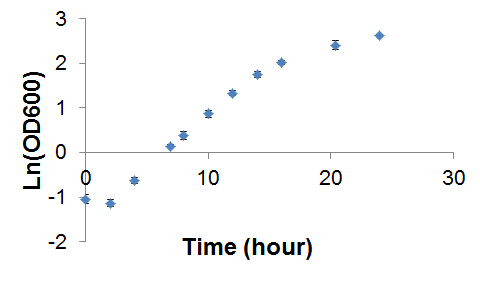

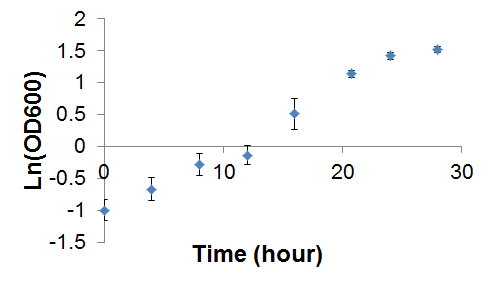


A

B

C

D

**Additional file 1: Fig. S1:** Pre-evaluation of mid-log phase for microplate cultivation. Microplate cultivation was performed by inoculating 5 μL over-night 20 g L^-1^ glucose pre-culture into 95 μL fresh YNB media with 20 g L^-1^ glucose (A), 20 g L^-1^ sucrose (B), 20 g L^-1^ galactose (C) or 2% v/v ethanol (D); Mean values ± standard deviations are shown from quadruplicate measurements.

*P_PDA1_*

*P_SSA1_*

*P_SSB1_*

*P_RPL8B_*

*P_RPL4_*

*P_YEF3_*

*P_RPL15A_*

*P_RPL3_*

*P_TEF2_*

*P_TEF1_*

*P_PGK1_*

*P_TDH3_*

**Additional file 1: Fig. S2:** Correlation of GFP fluorescence determination in the strains using either the destabilized GFP (yEGFP-*CLN2*_PEST_) or the normal GFP (yEGFP) as the reporter. The strains were cultivated on YNB media with 20 g L^-1^ glucose in microplates. Each data point represents the relative GFP fluorescence level (% auto-fluorescence) driven by different promoters, as annotated on the figure. N = 3 for each data point.

**Additional file 1: Fig. S3:** Post-hoc test for fluorescence levels (sorted from low to high) of various promoter-*yEGFP* strains on different carbon source. When Bartlett's test *p* value was > 0.05, Tukey’s test was used; else, Games-howell’s test was used. Ranges with *p* >0.05 were highlighted. The auto-fluorescence (Ref) was determined from the reference strain (ILHA GH4) in parallel.


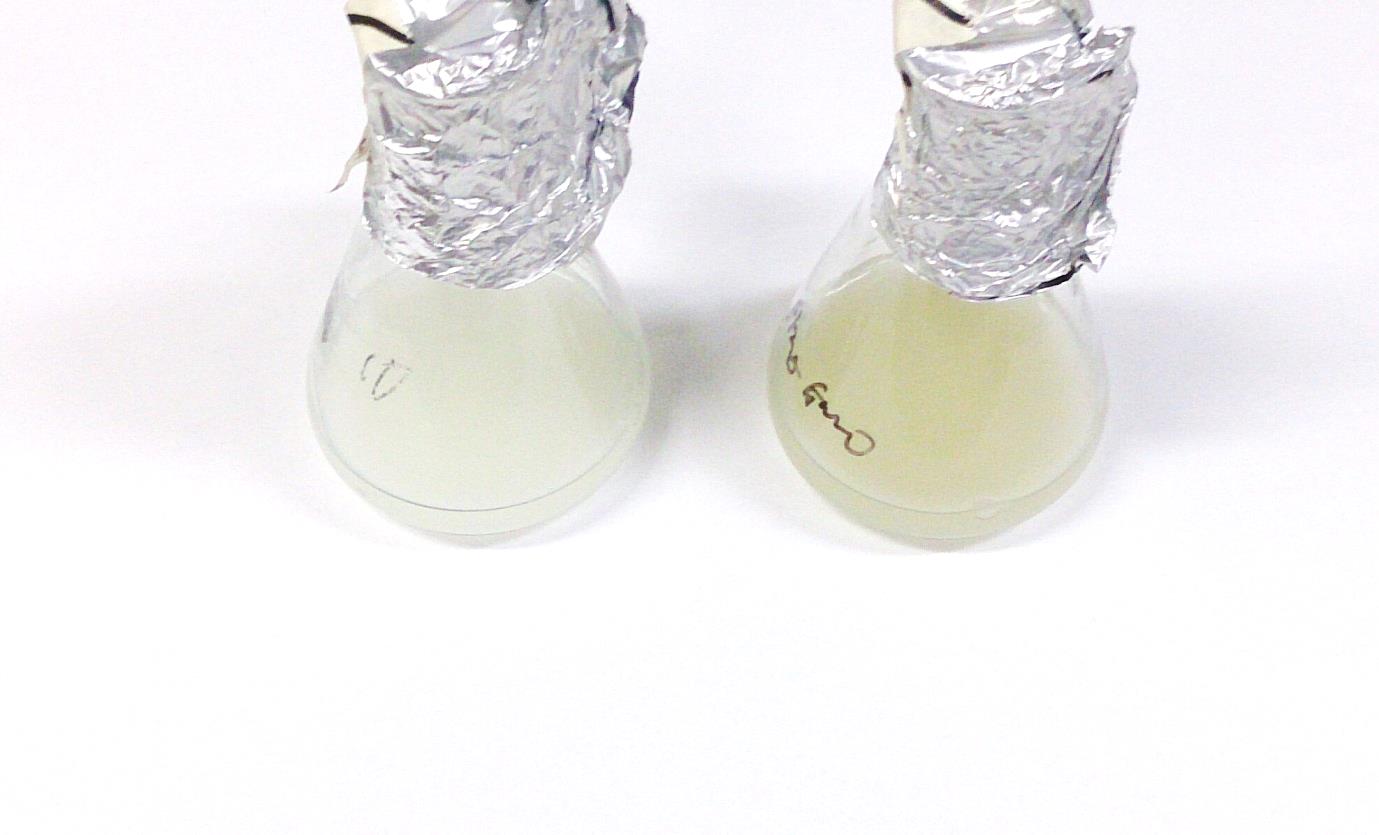


With copper

Without copper

**Additional file 1: Fig. S4:** Yeast cultures with/without copper addition.

A

C


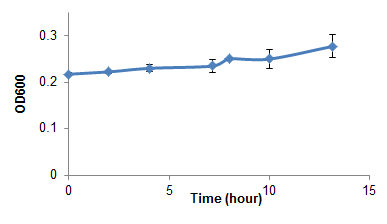


E

Log10(FL1.A/FSC.A)


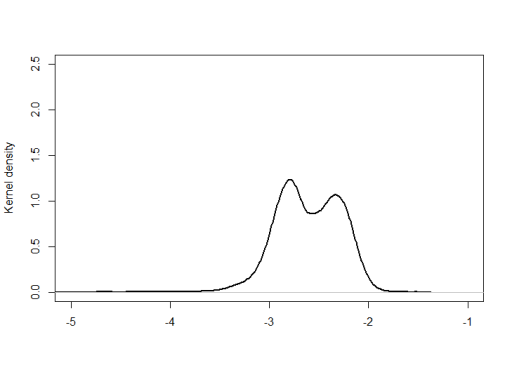

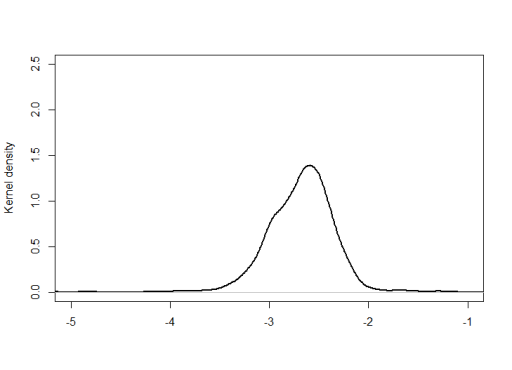


24 hour

36 hour

B

Log10(FL1.A/FSC.A)

-5 -4 -3 -2 -1

Kernel density

0 0.5 1.0 1.5 2.0 2.5

Kernel density

0 0.5 1.0 1.5 2.0 2.5


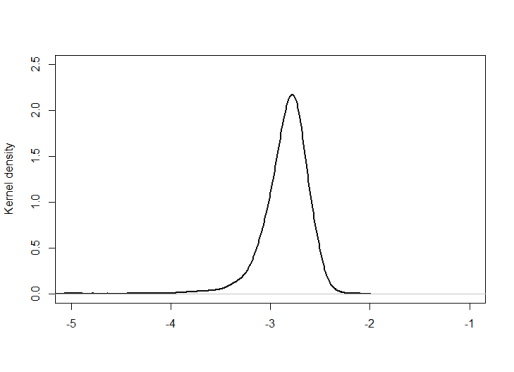

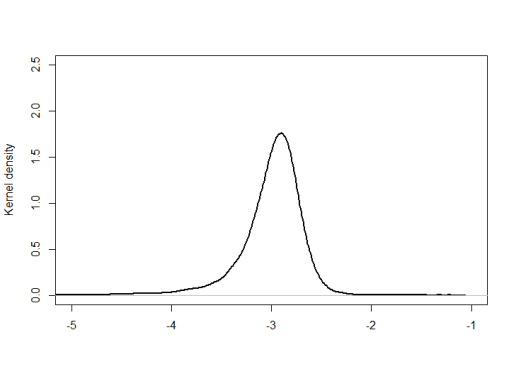


Log10(FL1.A/FSC.A)

24 hour

36 hour

-5 -4 -3 -2 -1

Kernel density

0 0.5 1.0 1.5 2.0 2.5

Kernel density

0 0.5 1.0 1.5 2.0 2.5


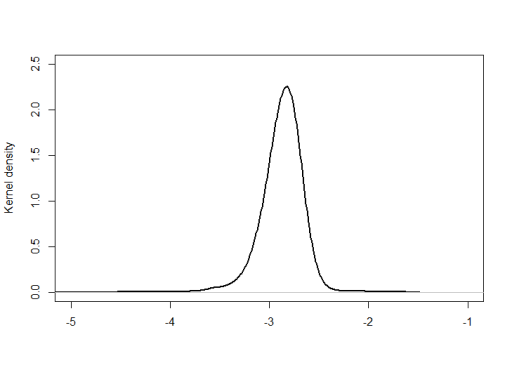

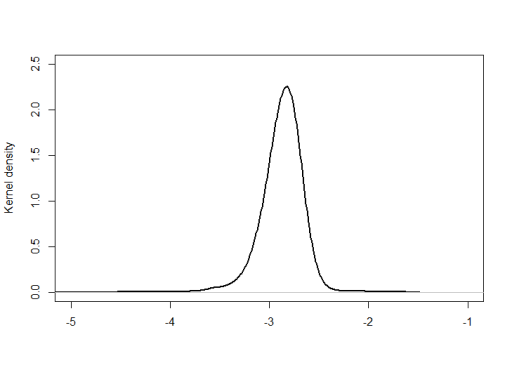

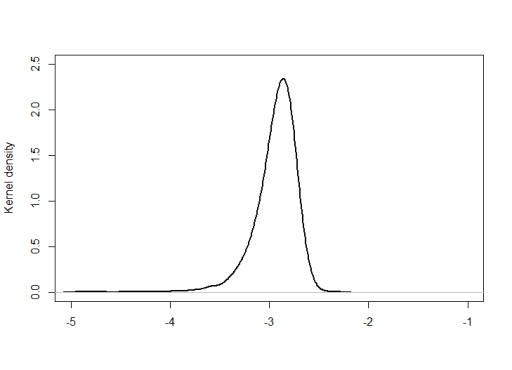


0 hour

2 hour

13 hour

D

Log10(FL1.A/FSC.A)

-5 -4 -3 -2 -1

Kernel density

0 0.5 1.0 1.5 2.0 2.5

Kernel density

0 0.5 1.0 1.5 2.0 2.5

Kernel density

0 0.5 1.0 1.5 2.0 2.5


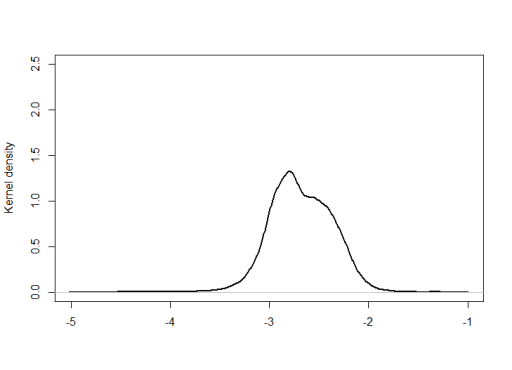

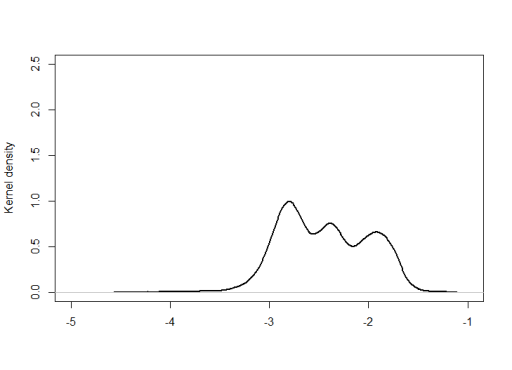

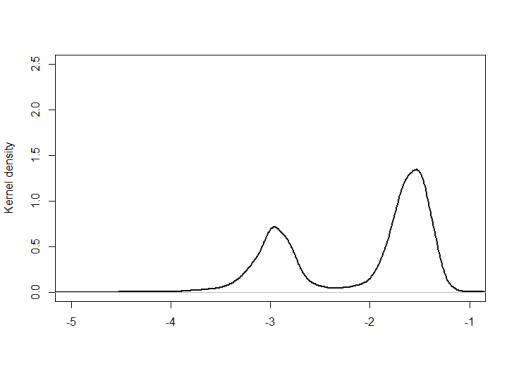


0 hour

2 hour

13 hour

-5 -4 -3 -2 -1

Kernel density

0 0.5 1.0 1.5 2.0 2.5

Kernel density

0 0.5 1.0 1.5 2.0 2.5

Kernel density

0 0.5 1.0 1.5 2.0 2.5

**Additional file 1: Fig. S5:** De-repression of *ADH2* promoter: A & B, batch cultivation of 20 g L^-1^ glucose (MES-buffered); C, D & E, batch cultivation of 2% v/v ethanol, initiated by inoculating 24 hour cultures from MES-buffered batch cultivation of 20 g L^-1^ glucose. A, C & E, the reference strain (ILHA GH4, without GFP); B & D, the strain (ILHA GH7S) with GFP controlled by *ADH2* promoter.
